# The Altitudinal Patterns of Leaf C∶N∶P Stoichiometry Are Regulated by Plant Growth Form, Climate and Soil on Changbai Mountain, China

**DOI:** 10.1371/journal.pone.0095196

**Published:** 2014-04-17

**Authors:** Ning Zhao, Nianpeng He, Qiufeng Wang, Xinyu Zhang, Ruili Wang, Zhiwei Xu, Guirui Yu

**Affiliations:** 1 Synthesis Research Center of Chinese Ecosystem Research Network, Key Laboratory of Ecosystem Network Observation and Modeling, Institute of Geographic Sciences and Natural Resources Research, Chinese Academy of Sciences, Beijing, China; 2 University of Chinese Academy of Sciences, Beijing, China; Lakehead University, Canada

## Abstract

Understanding the geographic patterns and potential drivers of leaf stoichiometry is critical for modelling the nutrient fluxes of ecosystems and to predict the responses of ecosystems to global changes. This study aimed to explore the altitudinal patterns and potential drivers of leaf C∶N∶P stoichiometry. We measured the concentrations of leaf C, N and P in 175 plant species as well as soil nutrient concentrations along an altitudinal transect (500–2300 m) on the northern slope of Changbai Mountain, China to explore the response of leaf C∶N∶P stoichiometry to plant growth form (PGF), climate and soil. Leaf C, N, P and C∶N∶P ratios showed significant altitudinal trends. In general, leaf C and C∶N∶P ratios increased while leaf N and P decreased with elevation. Woody and herbaceous species showed different responses to altitudinal gradients. Trees had the largest variation in leaf C, C∶N and C∶P ratios, while herbs showed the largest variation in leaf N, P and N∶P ratio. PGF, climate and soil jointly regulated leaf stoichiometry, explaining 17.6% to 52.1% of the variation in the six leaf stoichiometric traits. PGF was more important in explaining leaf stoichiometry variation than soil and climate. Our findings will help to elucidate the altitudinal patterns of leaf stoichiometry and to model ecosystem nutrient cycling.

## Introduction

Understanding the spatial patterns and the controlling factors for leaf C∶N∶P stoichiometry is critical for elucidating the patterns of nutrient fluxes across ecological gradients and the response of vegetation to global change [1]–[4]. The latitudinal patterns of leaf stoichiometry have been widely investigated at regional [5]–[9] and global scales [2], [10], and some general biogeographic patterns have been uncovered. However, variations in leaf stoichiometry along altitudinal gradients have met with ambiguous results. Some studies found that leaf C, N and P increase with elevation [11]–[14], but in other studies the opposite trend has also been found [15]–[20]. Previous studies on tropical, subtropical mountains and subarctic tundra suggest that leaf N and P declined with altitude [15]–[18]. In addition, non-linear relationships have been detected between leaf stoichiometric traits and altitude. For example, it is reported that leaf N and P at first increased and then decreased with increasing elevation in Mount Gongga, China and the Peruvian Andes [19]–[20]. Therefore, intensive studies are required to obtain a more general representation of the altitudinal patterns of leaf stoichiometry.

Plant growth form (PGF), climate and soil influence leaf C∶N∶P stoichiometry in a complex way [2], [21]–[23]. A series of hypotheses has been developed to interpret the geographic patterns and their underlying mechanisms, including the Plant Physiological Hypothesis [2], the Biogeochemical Hypothesis [2], and the Growth Rate Hypothesis [24]. The Plant Physiological Hypothesis suggests that plants increase leaf N and P content to offset the deceleration of plant metabolic rates caused by the low activity of enzymes at low temperatures [2]. The Biogeochemical Hypothesis assumes that soil nutrient availability, which is influenced by temperature and precipitation through organic matter decomposition and leaching effects, respectively, has a significant effect on leaf nutrient concentrations [2], [9], [22], [25]–[27]. The Growth Rate Hypothesis proposes that changes in plant growth rate bring a corresponding change in leaf stoichiometry. It claims that an elevated demand for P-rich ribosomal RNA under rapid growth drives variation in the P content and thus causes the corresponding change in the C∶P and N∶P ratios [24]. These hypotheses illustrate the factors driven by latitude that contribute to the biogeographic patterns of leaf stoichiometry. However, the montane landscape has its own unique features. For example, the environment and the vegetation type are known to change dramatically and rapidly with altitude, even over short distances. As we know, intrinsic to all mountainous areas are decreases in air temperature, soil nutriment availability and increases in relative humidity [28], which lead to more stressful environments for plants relative to lower altitudes. According to the Plant Physiological Hypothesis, plants should increase leaf N and P along elevation because of the reduced temperature. Whereas, to survival under low temperature stress and nutrient-limiting conditions plants tend to have lower growth rate [29]. So, it is more likely that leaf N and P decline while N∶P increase along elevation according to the prediction of the Biogeochemical Hypothesis and the Growth Rate Hypothesis. In fact, the discrepancy of previous results of altitudinal patterns suggests that the relationship between abiotic environmental conditions and leaf stoichiometry can vary among different regions or be impacted by different species composition, soil types or plant ecological strategies. Therefore, the relative effects of PGF, climate and soil on the altitudinal patterns of leaf stoichiometry need to be further understood.

Changbai Mountain is a volcanic mountain in northeastern China. The vegetation varies from broad-leaved forest at low elevation to alpine tundra at high elevation and has been deemed a mirror of the horizontal zonation of vegetation from temperate zones to frigid zones on the Eurasian continent [30]–[31]. In this study, we measured the concentrations of leaf C, N, and P in 175 plant species as well as the total and available N and P in the soil along an altitudinal transect (500–2300 m) on the northern slope of Changbai Mountain. The main objectives of this study were to (1) explore the altitudinal patterns of leaf stoichiometry; (2) compare the response of the leaf stoichiometric traits of different PGFs to the environmental gradient; and (3) determine the key factors controlling the altitudinal patterns of leaf stoichiometry.

## Materials and Methods

### Site description

Changbai Mountain (41°23'N–42°36'N, 126°55'E–129°00'E) is located in Jilin Province, northeastern China. It is the highest mountain in northeastern china and is the head of three large rivers (the Songhua River, the Yalu River and the Tumen River). The climate belongs to a temperate continental montane climate. As the elevation rises from 500 to 2744 m, the mean annual temperature (MAT) decreases from 3.5 to–7.4°C and the mean annual precipitation (MAP) increases from 720 to 1400 mm [32]. Changbai Mountain has obvious vertical vegetation zonation, including summer green broad-leaved forest (below 700 m), Korean pine and broad-leaved mixed forest (700–1100 m), dark-coniferous spruce and fir forest (1100–1800 m), sub-alpine birch forest (1800–2100 m), and alpine tundra (above 2100 m). Changbai Mountain is one of the few well-conserved natural ecosystems on Earth. All these factors make it an optimal site to investigate the altitudinal patterns of leaf stoichiometry.

### Sampling and measurements

**Figure 1:**
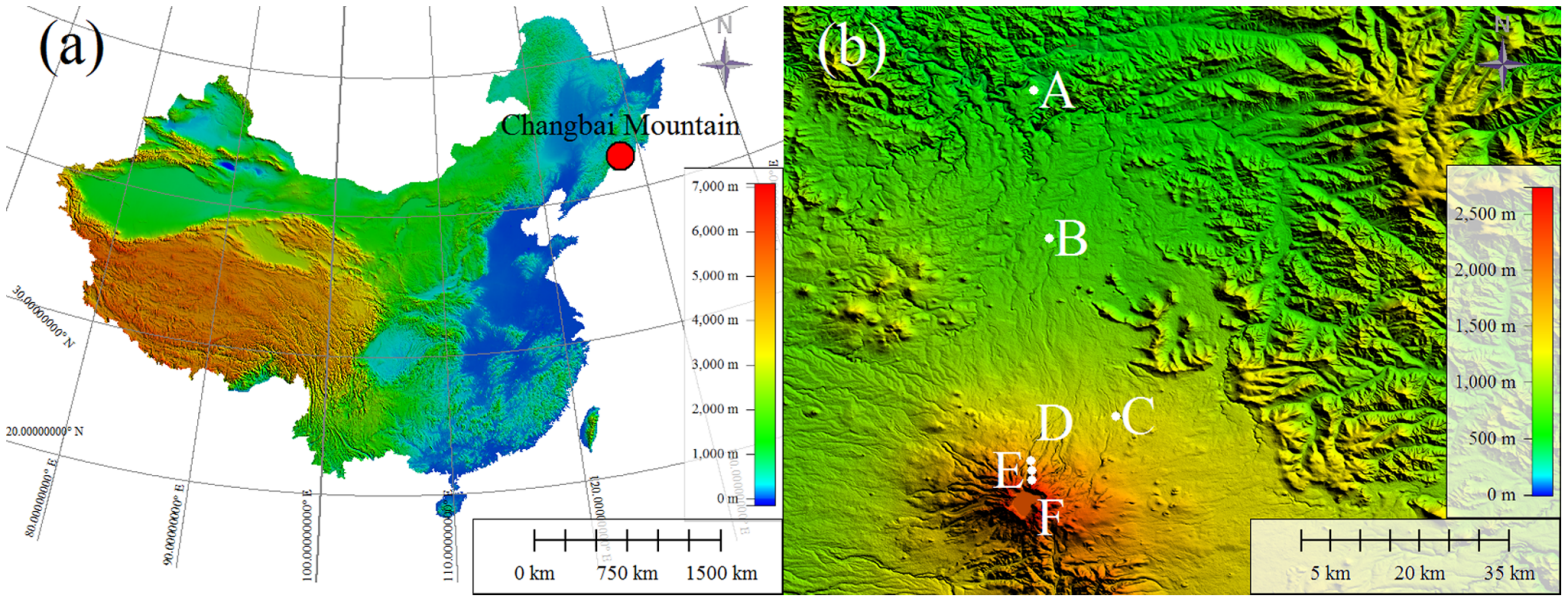
Experimental area and sampling sites along the northern slope of Changbai Mountain, China.

In early August 2012, we established six sampling sites (site A to site F) with the altitudinal gradient along the northern slope of Changbai Mountain. Locations and the main characteristics of sampling sites were showed in Fig. 1 and Table 1. Climatic data, such as mean annual temperature (MAT) and mean annual precipitation (MAP) were from literature (See Table 1 for more details) [33]. At each site, we set up four plots (30 m×40 m) where we collected leaves from the observed plant species and took soil samples. There was a total of 279 plant species collected across the six sites. Specifically, from site A to site F there was a distribution of 72, 91, 36, 38, 22, and 20 species, respectively (Table 1). If we consider the replication of plant species in the six sites, a total of 175 species belonging to 54 families were sampled from the six sites.

**Table 1:** Site descriptions of vegetation and soil properties.

Site	Vegetation type	Altitude(m)	Latitude	Longitude	Soil type	MAT(°C)	MAP(mm)	STC(mg g^−1^)	STN(mg g^−1^)	STP(mg g^−1^)	SAN(mg g^−1^)	SAP(mg g^−1^)	pH	No. ofSpecies
Site A	Broad-leaved forest	540	42°37'	128°4'	Albi-Boric Argosols	2.9	632	88.54	7.39	1.54	0.08	0.02	5.31	72
Site B	Mixed coniferous broad-leaved forest	753	42°24'	128°5'	Albi-Boric Argosols	2.6	691	60.62	4.92	1.36	0.07	0.01	5.01	91
Site C	Dark-coniferous spruce-fir forest	1286	42°8'	128°11'	Bori-UdicCambosols	0.3	811	15.79	0.78	0.42	0.04	0.02	5.23	36
Site D	Ermans birch forest	1812	42°4'	128°4'	Umbri-GelicCambosols	−2.3	967	54.11	3.79	0.93	0.07	0.01	5.03	38
Site E	Alpine tundra	2008	42°3'	128°3'	Permi-GelicCambosols	−3.3	1038	43.52	2.71	0.51	0.06	0.01	5.02	22
Site F	Alpine tundra	2357	42°2'	128°3'	Permafrost cold Cambisols	−4.8	1154	31.37	2.20	0.40	0.05	0.01	5.14	20

MAT, mean annual temperature; MAP, mean annual precipitation; STC, soil total carbon; STN, soil total nitrogen; STP, soil total phosphorus; SAN, soil available nitrogen; SAP, soil available phosphorus. MAT, MAP and soil type are derived from Shen et al. (2013) [33].

For each plant species, we collected sun-exposed and mature leaves (leaf blades for grasses) from five to ten individuals. Leaf samples were oven-dried at 60°C in the laboratory and were ground to a fine powder using a ball mill (MM400, Retsch, Germany) for chemical analysis. At each plot, soil samples were randomly collected from 30–50 points in the 0–10 cm and 10–30 cm layers using a soil sampler (diameter 6 cm), resulting in a mixed soil sampling (>5 kg) from each plot. The fresh soil samples were sieved through 2-mm meshes, and roots and visible organic debris were removed by hand. Approximately 100 g of each soil sample was air-dried in a ventilation room to analyse the soil properties (C and N, pH, and others). The remaining portion of each soil sample was stored at 4°C in refrigerators for available N and P analysis.

The total C and N concentrations of leaf and soil samples were determined by dry combustion using an elemental analyser (Vario MAX CN Elemental Analyzer, Elementar, Germany). Total P concentrations were measured by the ammonium molybdate method using a continuous-flow analyser (AutoAnalyzer3 Continuous-Flow Analyzer; Bran Luebbe, Germany) after H_2_SO_4_-HCLO_4_ digestion for leaves and H_2_SO_4_-H_2_O_2_-HF digestion for soil [34]–[35]. Soil inorganic N (NH_4_^+^-N and NO_3_^−^-N) in the filtrates was extracted using 2 mol L^−1^ KCl and determined using a continuous-flow analyser (AutoAnalyzer 3 Continuous-Flow Analyzer; Bran Luebbe, Germany) [36]. To measure available P, fresh soil samples were extracted using 0.5 mol L^−1^ NaHCO_3_, and the P concentration of the extract was determined by the ammonium molybdate method. Soil pH was determined with a pH meter using soil mixed with distilled water (ratio 1∶2.5). For each soil variable, the value used here was the average for the two depths in each plot.

### Ethics statement

Our field studies obtained special permission from Changbai Mountain National Reserve, Jilin Province, China. We have no commercial interests or conflicts of interest in performing this work.

### Data analysis

The data were explored at the species level and the site-species level, respectively. At the species level, we statistically summarised the means and the coefficients of variation (CV) of the leaf C, N, P, and C∶N∶P ratios. Furthermore, all plant species were divided into three PGFs: herbs, shrubs, and trees. Differences in leaf stoichiometric traits among different PGFs were tested using analysis of variance (ANOVA) with the Duncan *post hoc* tests of significance.

At the site-species level, the relationships between leaf stoichiometric traits and altitude were explored by linear regressions after the leaf C, N, P, and C∶N∶P ratios were log-transformed to normalise the distributions. Furthermore, we compared the relationship between the leaf C, N, P and C∶N∶P ratios and altitude among PGFs in order to examine the responses of different PGFs to environmental gradients (Table S1).

The effects of PGF, climate and soil on the leaf C, N, P and C∶N∶P ratios were tested using the General Linear Model (GLM). As a first step, within climate (MAP and MAT) and soil variables (soil total carbon, STC; soil total nitrogen, STN; soil total phosphorus, STP; soil available nitrogen, SAN; soil available phosphorus, SAP and pH), stepwise selection of variables was performed to exclude variables that did not contribute significantly (*P*<0.01) to the explained variation (For full details see Table S2, S3, S4). Then, the partial General Liner Model (partial GLM) separated the variance explained by different factors into the independent effects of each individual factor and the interactive effects between factors (Table S5) (for details, see Heikkinen et al. 2005) [37].

All statistical analyses were performed with R 2.15.2 [38].

## Results

### Statistics of leaf C, N, P and C∶N∶P ratios

Leaf C, N, P and the C∶N∶P ratios of the plants on Changbai Mountain varied greatly with a range of 246.5–549.2 mg g^−1^ for C, 10.2–46.9 mg g^−1^ for N, and 0.8–5.5 mg g^−1^ for P. The C∶N∶P ratios ranged from 8.0 to 45.7 for C∶N, 79.8 to 645.3 for C∶P, and 3.4 to 24.8 for N∶P. Leaf C, N, P and the C∶N∶P ratios varied 2 to 8 fold across species. Among leaf C, N, and P concentrations, leaf P had the greatest variation with a CV of 0.35, whereas leaf C had the smallest variation (CV = 0.08), which resulted in C∶P having the greatest variation (CV = 0.43) and N∶P having the smallest (CV = 0.28) (Table 2).

**Table 2:** Leaf C, N, P and C∶N∶P ratios for plant species on the Changbai Mountain, northeast China.

		C (mg g^−1^)		N (mg g^−1^)		P (mg g^−1^)		C∶N ratio		C∶P ratio		N∶P ratio	
	n	Mean	CV	Mean	CV	Mean	CV	Mean	CV	Mean	CV	Mean	CV
Herbs	105	422.98^a^ † ‡	0.07	25.13^a^	0.27	2.49^a^	0.30	18.42^a^	0.29	187.24^a^	0.34	10.54^a^	0.24
Shrubs	37	457.07^b^	0.07	21.98^b^	0.25	1.84^b^	0.44	22.53^b^	0.27	286.58^b^	0.41	13.01^b^	0.31
Trees	33	466.88^b^	0.06	22.92^b^	0.26	1.79^b^	0.24	22.29^b^	0.34	283.17^b^	0.35	13.11^b^	0.23
All species	175	438.56	0.08	24.13	0.27	2.22	0.35	19.71	0.32	226.33	0.43	11.54	0.28

† Mean values and the coefficient of variation (CV) are reported, along with the number of samples (n).

‡ Differences among PGFs were tested using ANOVA with Duncan post hoc tests; different superscript letters (a and b) in each column indicate significant differences in the mean values at *P*<0.05.

Leaf C, N, P and the C∶N∶P ratios differed significantly among different PGFs (Table 2). Leaf N and P were remarkably higher in herbaceous species than in woody species, whereas leaf C and C∶N∶P ratios were higher in trees and shrubs than in herbs (Table 2).

### Patterns of leaf C, N, P and C∶N∶P ratios along altitudinal gradients

**Figure 2:**
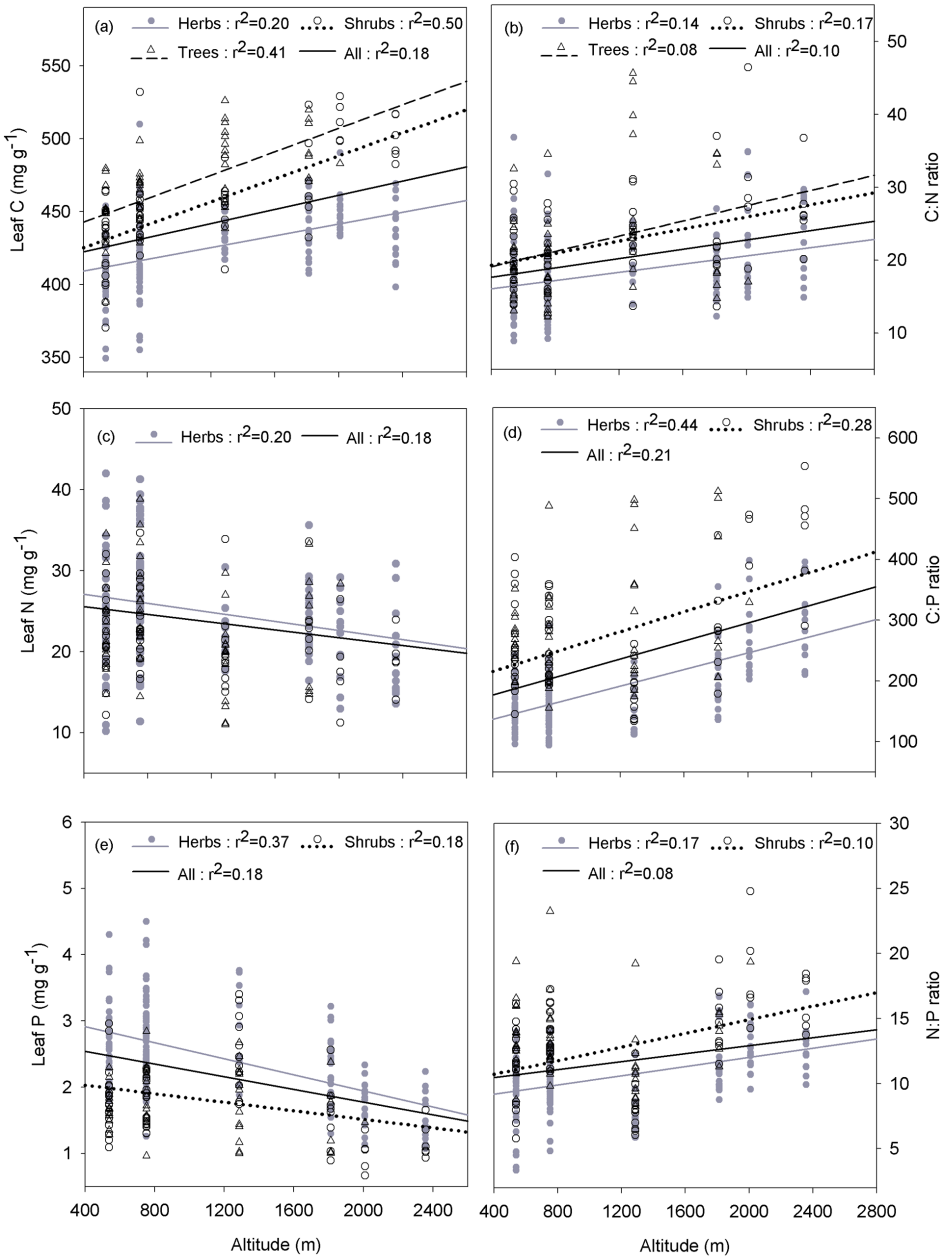
Changes in leaf C, N, P and C∶N∶P ratios with the altitudinal gradient. Lines are plotted if regressions were significant at *P*<0.05. Note log scale used on y-axis.

For all plant species, leaf C, N, P and the C∶N∶P ratios were significantly correlated with altitude (*P*<0.001) (Fig. 2). Leaf C increased significantly along elevation (r^2^ = 0.18, *P*<0.001) (Fig. 2a), but leaf N and P decreased with increased altitude (Fig. 2c, e). Meanwhile, the leaf C∶N∶P ratios were positively correlated with altitude (Fig. 2b, d, f).

The leaf stoichiometric traits of different PGFs showed different responses to altitudinal gradients. Leaf C exhibited a remarkable increase along elevation for trees (*r^2^* = 0.41, *P*<0.001), shrubs (*r^2^* = 0.50, *P*<0.001), and herbs (*r^2^* = 0.20, *P*<0.001) (Fig. 2a). Leaf N was negatively correlated with altitude for herbs (*r^2^* = 0.07, *P*<0.001) (Fig. 2c) but showed no significant change for trees and shrubs. For shrubs and herbs, leaf P decreased significantly with increasing altitude (Fig. 2e).

### Effects of PGF, climate and soil on leaf stoichiometric traits

**Figure 3:**
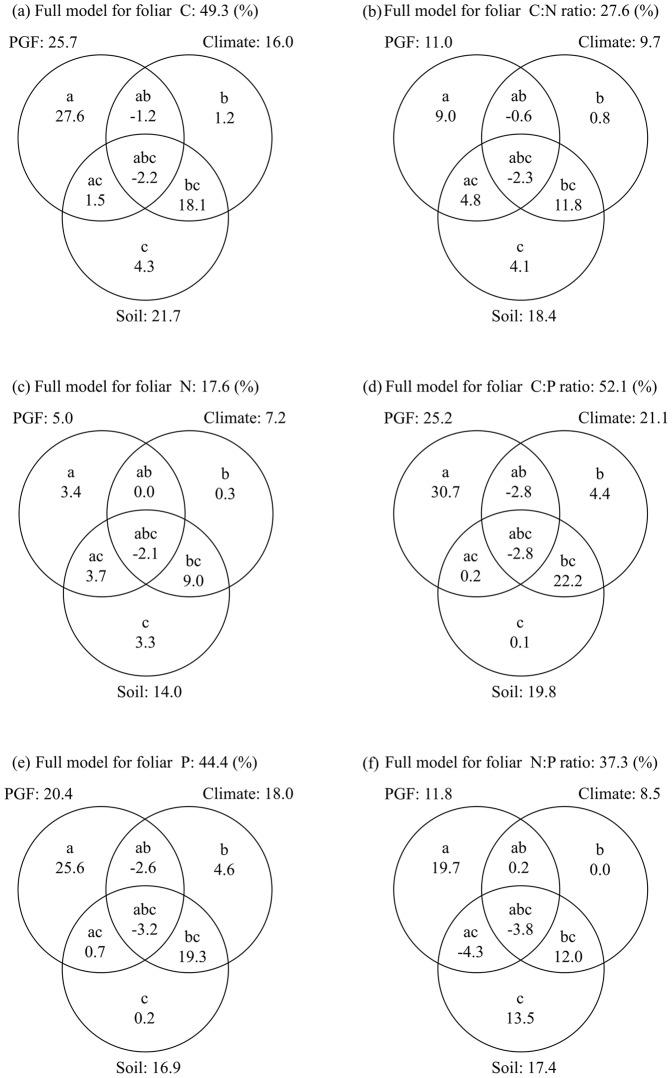
Variation partitioning (*R^2^*, %) of PGF, climate and soil in accounting for the variations in leaf C, N, P and C∶N∶P ratios. *a, b,* and *c* denote the independent effect of plant growth form (PGF), climate and soil, respectively; *ab, ac*, and *bc* are the interactive effect between PGF and climate, PGF and soil, climate and soil, respectively; *abc* denotes the interactive effect among the three factors.

The general linear model (GLM) showed that PGF, climate and soil together explained 17.6% to 52.1% of the variation in the six leaf stoichiometric traits (Fig. 3). The independent effect of PGF (*a*) showed the largest contribution (3.4–30.7%). The interactive effect of soil and climate (*bc*) also had substantial contributions (9.0–22.2%), although the independent effect of each factor was small. The total effect of PGF (*a+ab+ac+abc*) accounted for the largest contribution to the variations in leaf C and P concentrations and the C∶P ratio (explained 25.7%, 20.4% and 25.2%, respectively), while the total effect of soil (*c+ac+bc+abc*) most contributed to leaf N and the C∶N and N∶P ratios (explained 14.0%, 18.4% and 17.4%, respectively).

## Discussion

### Variations of leaf C, N, P and C∶N∶P ratios

The average of leaf N, P and the C∶N∶P ratios of the 175 plant species on Changbai Mountain were different from those reported at regional scale, whereas leaf C showed no remarkable distinction. Our results showed that the mean of leaf C on Changbai Mountain was 438.56 mg g^−1^, which is nearly equivalent with that of 213 species across the Chinese grassland (438 mg g^−1^), as reported by He et al. (2006). However, the means of leaf N and P were 20.9% and 57.5% higher than those of the Chinese flora (19.96 and 1.41 mg g^−1^ for leaf N and P, respectively), while the N∶P ratio was 19.0% lower (the N∶P ratio was 14.2 for the Chinese flora) [23]. The higher concentrations of leaf N and P on Changbai Mountain may be attributed to the soil fertility being higher than the mean level of N and P storage in China [22], [39], with the STN at 3.63 mg g^−1^ and the SAN at 0.06 mg g^−1^. Similarly, the STP of Changbai Mountain (0.86±0.03 mg g^−1^) was higher than the average of China (0.56 mg g^−1^) [7].

We found that leaf P was more variable than leaf C and N, which is consistent with previous results at different spatial scales [8], [40]–[42]. Our findings support the Stability of Limiting Elements Hypothesis to some extent, which suggests that nutrients required in a high concentration in plants and considered most frequently limiting in environment should show a small variation in their concentration and lower sensitivity to the environmental factors [22], [43]. It implies that leaf P is less stable and has weaker stoichiometric homeostasis than leaf N. When comparing C∶N∶P ratios, the variation was largest for the C∶P ratio and smallest for the N∶P ratio (Table 2). The larger variation in the C∶P ratio was mainly due to the large variation in P. In contrast, the N∶P ratio varied less than N or P alone because of the close relationship in biochemical reaction between N and P.

### Altitudinal patterns of leaf C, N, P and C∶N∶P ratios

Leaf C, N, P and C∶N∶P ratios exhibited remarkable altitudinal patterns on Changbai Mountain. Leaf C increased significantly with altitude, in agreement with the results of a meta-analysis conducted on global mountain ranges [13]. The increased leaf C concentration was most likely caused by an increase in non-structural C (NSC), including starch, low molecular weight sugars and storage lipids. Plant species at higher elevations experience more stressed environments than do those at lower elevations; thus, higher NSC concentrations accumulated to balance the osmotic pressure of cells and to resist freezing [13], [44]–[45].

Our results support the pattern that leaf N and P declined with elevation, consisting with results of some previous studies [15]–[18]. Meanwhile, the increased leaf N∶P ratio was also found in studies conducted on subarctic tundra in northern Sweden and northwestern Himalaya [14], [18], although other studies found different tendency [12], [17], [20]. The deceleration of plant metabolic rates and the limitation of nutrient availability could interact to shape the altitudinal patterns of leaf N, P and N∶P ratio. Some studies have demonstrated that low temperatures can limit soil microbe activity, resulting in low decomposition of organic matter and available nutrients in soil, and thus depress nutrient uptake by roots [2], [25]. Furthermore, aggravated soil leaching caused by the increased precipitation at higher elevations deteriorates nutrient availability [46]. In fact, a soil fertility decrease along the altitudinal gradient was clearly observed on Changbai Mountain (Fig. S1). Therefore, declined metabolic rates and nutrient limitation together shaped the pattern that leaf N and P declined with elevation. The increased N∶P ratio suggest that N concentrations decrease slower than P concentrations. Moreover, to survival under low temperature stress, plants tend to have lower growth rate which might lead to higher N∶P ratio. As was mentioned above, the altitudinal patterns are consistent with the prediction of the Biogeochemical Hypothesis and the Growth Rate Hypothesis. It is possible to assume that, these hypotheses which based on latitudinal patterns can also well describe the drivers of altitudinal patterns.

### The response of different plant growth forms

Different plant growth forms respond differently to environmental gradients for leaf stoichiometric traits. All the observed species were divided into three PGFs (trees, shrubs and herbs), and linear regressions of leaf stoichiometric traits against altitude were then performed to compare the response of different PGFs. The regression slopes indicate the degree of variation for leaf stoichiometric traits of different PGFs. A steeper slope indicates greater variation of leaf stoichiometric traits along elevation. Among the three PGFs, trees showed the steepest slope for leaf C, corresponding with the largest variation for the C∶N and C∶P ratios (Fig. 2, Table S1), while leaf N and P showed no significant change with increasing altitude. In contrast, herbs showed the largest variation in leaf N, P and the N∶P ratio (Fig. 2, Table S1). The different responses may be influenced by the distinct life strategies exhibited by different PGFs. Slow-growing woody species are likely to be competitors or stress tolerators (*C* or *S* selected) with relatively conservative stoichiometric traits [47]. However, fast-growing herbaceous species, which adopt a ruderal strategy (*R*-selected), exhibit more flexibility for leaf stoichiometric traits. The more dramatic increase in leaf C in trees might be due to the tree experiencing critically low temperatures at lower elevation and latitude than smaller plants [48]. Thus, as we mentioned before, increased leaf C would help trees to resist freezing [13].

### The relative effects of PGF, climate and soil

PGF, climate and soil jointly influenced the altitudinal patterns of leaf stoichiometry. GLM models were able to determine the explanatory capacity of PGF (*a*), climate (*b*) and soil (*c*) for different stoichiometric traits along the elevation gradient on Changbai Mountain. PGF (*a+ab+ac+abc*) accounted for the largest variations in leaf C, P, and the C∶P ratio, while soil (*c+ac+bc+abc*) was better able to explain the variation of leaf N and the C∶N and N∶P ratios (Fig. 3, Table. S5). However, the collinearity among PGF, climate and soil may obscure their true explanatory capacity. The partial GLM provided a better way to divide the total effect into independent and interactive effects [37]. Our results showed that the independent effect of PGF (*a*) was the largest contributor to the explained variations in leaf C, N, P and C∶N∶P ratios. In contrast, the independent effects of climate (*b*) and soil (*c*) were small (Fig. 3, Table S5). Our results are different from recent studies carried out on forest and shrub biomes at regional scale. Chen et al. (2011) and Liu et al. (2012) reported that the independent effect of PGF had the largest contribution to leaf N variation, while environmental factors and PGF were both important for leaf P and the N∶P ratio. A potential cause for such an inconsistency may be the different nutrient limitations in the different regions [8], [49]. Plants are most likely P-limited in most regions of China [7], whereas the vegetation of Changbai Mountain is mainly limited by N (Table S6). The difference in nutrient limitations in different regions may obscure the roles of other factors. Overall, these results suggest that the relationships between leaf C∶N∶P stoichiometry and environment, depending on the most limiting factor, should be different in different regions.

The effects of environmental factors, PGF and interactions between them on stoichiometric traits have received much less attention in previous studies. In spite of the small independent effect, the interactive effect of climate and soil (*bc*) was the largest explanatory factor among the interactions for all six stoichiometric traits (Fig. 3, Table S5). This strong interaction of climate and soil may be the cause of the obvious change in soil fertility along the altitudinal gradient (Fig. S1). Note that the interactive effects were negative when the relationship between two factors is mainly suppressive rather than additive [22], [37].

## Concluding Remarks

This study comprehensively characterised the relative effects of PGF, climate and soil on the altitudinal patterns of leaf C∶N∶P stoichiometry on Changbai Mountain, China. The altitudinal patterns of leaf stoichiometry were influenced by PGF, climate, soil and their interactions. In general, PGF has a stronger influence than soil and climate. Overall, the relationships between leaf C∶N∶P stoichiometry and environment, depending on the most limiting factor, should be different in different regions.

## Supporting Information

Figure S1 **Changes in soil nutrient and pH value with the altitudinal gradient on Changbai Mountain.** STC, soil total carbon; STN, soil total nitrogen; STP, soil total phosphorus; SAN, soil available nitrogen; SAP, soil available phosphorus. Error bars mean standard errors (SE) of variables.
(TIF)

Table S1 **Linear regressions of leaf stoichiometric traits on altitude for different plant growth forms.** “***” denotes P<0.001, “*” denotes P<0.05. Note log scale used on y-axis.
(DOCX)

Table S2 **Correlations between soil and environmental variables.** MAT, mean annual temperature; MAP, mean annual precipitation; STC, soil total carbon; STN, soil total nitrogen; STP, soil total phosphorus; SAN, soil available nitrogen; SAP, soil available phosphorus. Pearson coefficients in bold and with an asterisk indicate the correlation is significant at P<0.05.
(DOCX)

Table S3 **Model summary for the stepwise multiple regressions of leaf stoichiometric traits on MAP and MAT.** The variable that do not contribute significantly (P<0.01) to the explained variation will be excluded from the partial General Linear Models (partial GLM). MAT, mean annual temperature; MAP, mean annual precipitation.
(DOCX)

Table S4 **Model summary for the stepwise multiple regressions of leaf stoichiometric traits on soil variables.** The variables that do not contribute significantly (P<0.01) to the explained variation will be excluded from the partial General Linear Models (partial GLM). STC, soil total carbon; STN, soil total nitrogen; STP, soil total phosphorus; SAN, soil available nitrogen; SAP, soil available phosphorus.
(DOCX)

Table S5 **Summary of the partial General Linear Models (partial GLM) for the effects of PGF, climate and soil nutrient on leaf stoichiometric traits.** a, b, and c denote the independent effect of plant growth form (PGF), climate and soil, respectively; ab, ac, and bc are respectively the interactive effect between PGF and climate, PGF and soil, climate and soil; abc denotes the interactive effect among the three factors.
(DOCX)

Table S6 **Nutrition limitations of different vegetation types on Changbai Mountain.** Nutrient limitations is diagnosed following criteria proposed by Koerselman (1996): If N∶P ratio >16, plant growth is limited by P availability. If N∶P ratio <14, plant growth is limited by N availability. If N∶P ratio between 14 and 16, plant growth is co-limited by N and P together. “N” indicates N limitation; “N and P” indicates N and P co-limitation.
(DOCX)
