# Supplementary figures and images for: The Altitudinal Patterns of Leaf C∶N∶P Stoichiometry Are Regulated by Plant Growth Form, Climate and Soil on Changbai Mountain, China
Source: PLoS One. 2014 Apr 17;9(4):e95196. doi: 10.1371/journal.pone.0095196 (PMC3990608; doi:10.1371/journal.pone.0095196)

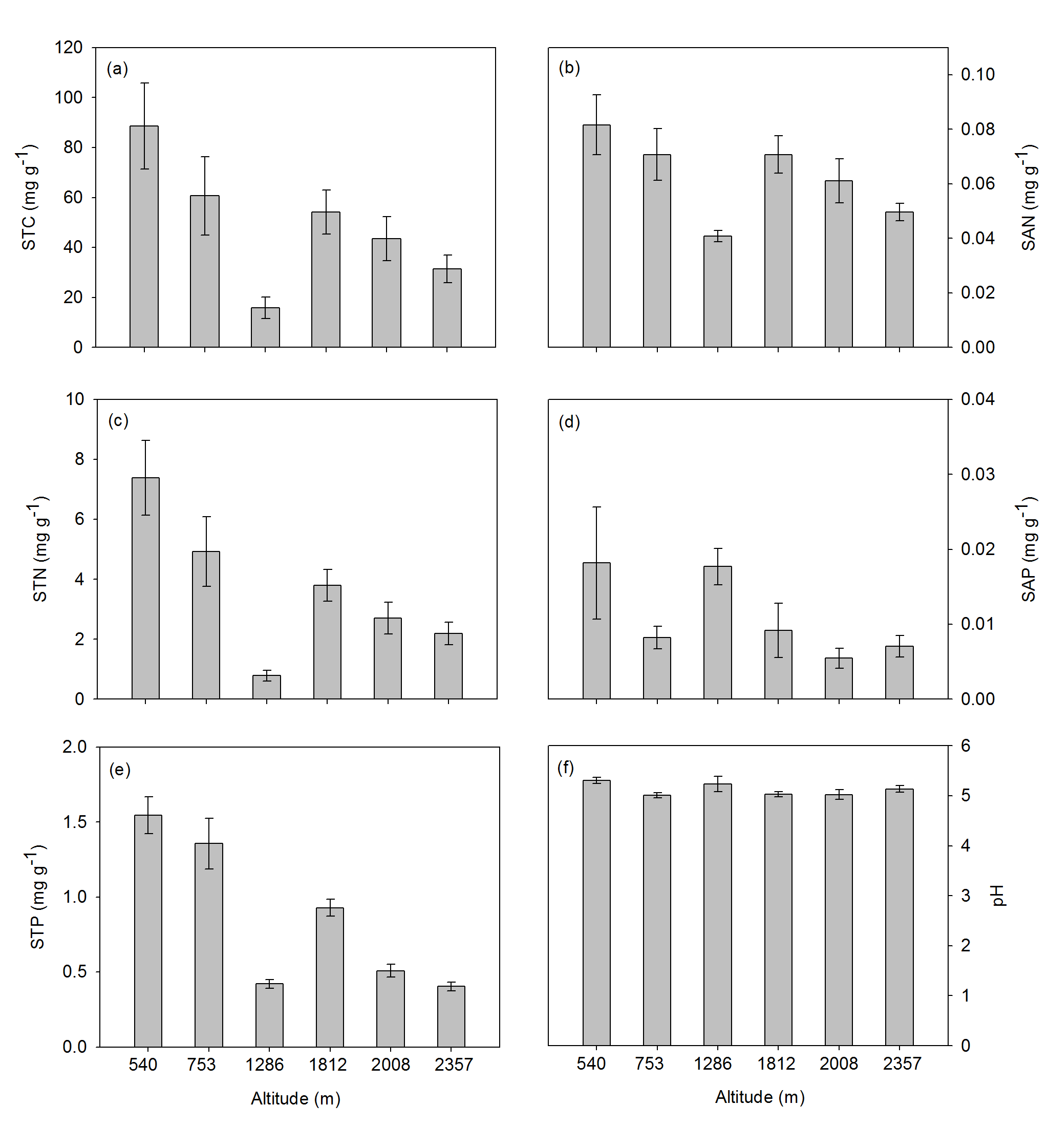

Supplement: Figure S1 — Changes in soil nutrient and pH value with the altitudinal gradient on Changbai Mountain. STC, soil total carbon; STN, soil total nitrogen; STP, soil total phosphorus; SAN, soil available nitrogen; SAP, soil available phosphorus. Error bars mean standard errors (SE) of variables. (TIF) [file pone.0095196.s001.tif]
