# Supplementary material for: The Altitudinal Patterns of Leaf C∶N∶P Stoichiometry Are Regulated by Plant Growth Form, Climate and Soil on Changbai Mountain, China
Source: PLoS One. 2014 Apr 17;9(4):e95196. doi: 10.1371/journal.pone.0095196 (PMC3990608; doi:10.1371/journal.pone.0095196)
Supplement: Table S1 — Linear regressions of leaf stoichiometric traits on altitude for different plant growth forms. “***” denotes P<0.001, “*” denotes P<0.05. Note log scale used on y-axis. (DOCX) [file pone.0095196.s002.docx]

**Table S1** Linear regressions of leaf stoichiometric traits on altitude for different plant growth forms. “***” denotes *P* < 0.001, “*” denotes *P* < 0.05. Note log scale used on y-axis

| PGF |  | Slope | Intercept | *r^2^* | *P* |  |
| --- | --- | --- | --- | --- | --- | --- |
| Herbs | C | 2.08E-05 | 2.60 | 0.195 | 5.11E-09 | *** |
|  | N | -4.72E-05 | 1.44 | 0.068 | 8.53E-04 | *** |
|  | P | -1.23E-04 | 0.52 | 0.371 | <2e-16 | *** |
|  | C:N | 6.80E-05 | 1.17 | 0.135 | 1.81E-06 | *** |
|  | C:P | 1.44E-04 | 2.09 | 0.444 | <2e-16 | *** |
|  | N:P | 7.57E-05 | 0.92 | 0.171 | 5.41E-08 | *** |
| Shrubs | C | 3.72E-05 | 2.61 | 0.502 | 8.08E-11 | *** |
|  | N | -3.85E-05 | 1.37 | 0.056 | 6.09E-02 |  |
|  | P | -1.12E-04 | 0.36 | 0.183 | 4.72E-04 | *** |
|  | C:N | 7.57E-05 | 1.25 | 0.169 | 8.21E-04 | *** |
|  | C:P | 1.49E-04 | 2.25 | 0.260 | 1.96E-05 | *** |
|  | N:P | 7.34E-05 | 1.00 | 0.104 | 1.00E-02 | * |
| Trees | C | 3.73E-05 | 2.63 | 0.405 | 1.36E-07 | *** |
|  | N | -5.05E-05 | 1.39 | 0.034 | 1.73E-01 |  |
|  | P | -3.77E-05 | 0.28 | 0.021 | 2.82E-01 |  |
|  | C:N | 8.79E-05 | 1.24 | 0.084 | 2.98E-02 | * |
|  | C:P | 7.50E-05 | 2.36 | 0.069 | 5.08E-02 |  |
|  | N:P | -1.29E-05 | 1.12 | 0.004 | 6.58E-01 |  |
