# Supplementary material for: The Altitudinal Patterns of Leaf C∶N∶P Stoichiometry Are Regulated by Plant Growth Form, Climate and Soil on Changbai Mountain, China
Source: PLoS One. 2014 Apr 17;9(4):e95196. doi: 10.1371/journal.pone.0095196 (PMC3990608; doi:10.1371/journal.pone.0095196)
Supplement: Table S2 — Correlations between soil and environmental variables. MAT, mean annual temperature; MAP, mean annual precipitation; STC, soil total carbon; STN, soil total nitrogen; STP, soil total phosphorus; SAN, soil available nitrogen; SAP, soil available phosphorus. Pearson coefficients in bold and with an asterisk indicate the correlation is significant at P<0.05. (DOCX) [file pone.0095196.s003.docx]

**Table S2** Correlations between soil and environmental variables. MAT, mean annual temperature; MAP, mean annual precipitation; STC, soil total carbon; STN, soil total nitrogen; STP, soil total phosphorus; SAN, soil available nitrogen; SAP, soil available phosphorus. Pearson coefficients in **bold** and with an asterisk indicate the correlation is significant at *P* < 0.05

|  | Altitude | MAT | MAP | STC | STN | STP | SAN | SAP |
| --- | --- | --- | --- | --- | --- | --- | --- | --- |
| MAT | **-0.996*** |  |  |  |  |  |  |  |
| MAP | **0.997*** | **-0.997*** |  |  |  |  |  |  |
| STC | -0.614 | 0.562 | -0.581 |  |  |  |  |  |
| STN | -0.671 | 0.621 | -0.635 | **0.992*** |  |  |  |  |
| STP | -0.623 | 0.796 | -0.799 | **0.920*** | **0.944*** |  |  |  |
| SAN | -0.534 | 0.488 | -0.507 | **0.973*** | **0.946*** | **0.894*** |  |  |
| SAP | -0.650 | 0.642 | -0.675 | 0.171 | 0.230 | 0.305 | 0.031 |  |
| PH | -0.393 | 0.362 | -0.392 | 0.128 | 0.192 | 0.128 | -0.071 | **0.874*** |
