# Supplementary material for: The Altitudinal Patterns of Leaf C∶N∶P Stoichiometry Are Regulated by Plant Growth Form, Climate and Soil on Changbai Mountain, China
Source: PLoS One. 2014 Apr 17;9(4):e95196. doi: 10.1371/journal.pone.0095196 (PMC3990608; doi:10.1371/journal.pone.0095196)
Supplement: Table S3 — Model summary for the stepwise multiple regressions of leaf stoichiometric traits on MAP and MAT. The variable that do not contribute significantly (P<0.01) to the explained variation will be excluded from the partial General Linear Models (partial GLM). MAT, mean annual temperature; MAP, mean annual precipitation. (DOCX) [file pone.0095196.s004.docx]

**Table S3** Model summary for the stepwise multiple regressions of leaf stoichiometric traits on MAP and MAT. The variable that do not contribute significantly (*P* < 0.01) to the explained variation will be excluded from the partial General Linear Models (partial GLM). MAT, mean annual temperature; MAP, mean annual precipitation

|  | Adjust *R^2^* | Contribution of the individual predictor (%) | |
| --- | --- | --- | --- |
|  | Full model | MAP | MAT |
| C | 0.164 |  | 100.0 |
| N | 0.072 | 77.1 | 23.0 |
| P | 0.181 | 100.0 |  |
| C:N | 0.104 | 100.0 |  |
| C:P | 0.211 | 100.0 |  |
| N:P | 0.091 |  | 100.0 |
