# Supplementary material for: The Altitudinal Patterns of Leaf C∶N∶P Stoichiometry Are Regulated by Plant Growth Form, Climate and Soil on Changbai Mountain, China
Source: PLoS One. 2014 Apr 17;9(4):e95196. doi: 10.1371/journal.pone.0095196 (PMC3990608; doi:10.1371/journal.pone.0095196)
Supplement: Table S4 — Model summary for the stepwise multiple regressions of leaf stoichiometric traits on soil variables. The variables that do not contribute significantly (P<0.01) to the explained variation will be excluded from the partial General Linear Models (partial GLM). STC, soil total carbon; STN, soil total nitrogen; STP, soil total phosphorus; SAN, soil available nitrogen; SAP, soil available phosphorus. (DOCX) [file pone.0095196.s005.docx]

**Table S4** Model summary for the stepwise multiple regressions of leaf stoichiometric traits on soil variables. The variables that do not contribute significantly (*P* < 0.01) to the explained variation will be excluded from the partial General Linear Models (partial GLM). STC, soil total carbon; STN, soil total nitrogen; STP, soil total phosphorus; SAN, soil available nitrogen; SAP, soil available phosphorus

|  | Adjust *R^2^* | Contribution of the individual predictor (%) | | | | | |
| --- | --- | --- | --- | --- | --- | --- | --- |
|  | Full model | STC | STN | STP | SAN | SAP | PH |
| C | 0.217 |  |  | 93.2 |  |  | 6.8 |
| N | 0.14 |  |  | 64.2 |  |  | 35.8 |
| P | 0.169 |  |  | 57.2 | 42.8 |  |  |
| C:N | 0.184 |  |  | 84.3 |  |  | 15.7 |
| C:P | 0.198 |  |  | 68.4 | 31.6 |  |  |
| N:P | 0.174 |  |  |  |  | 77.8 | 22.2 |
