# Supplementary material for: The Altitudinal Patterns of Leaf C∶N∶P Stoichiometry Are Regulated by Plant Growth Form, Climate and Soil on Changbai Mountain, China
Source: PLoS One. 2014 Apr 17;9(4):e95196. doi: 10.1371/journal.pone.0095196 (PMC3990608; doi:10.1371/journal.pone.0095196)
Supplement: Table S5 — Summary of the partial General Linear Models (partial GLM) for the effects of PGF, climate and soil nutrient on leaf stoichiometric traits. a, b, and c denote the independent effect of plant growth form (PGF), climate and soil, respectively; ab, ac, and bc are respectively the interactive effect between PGF and climate, PGF and soil, climate and soil; abc denotes the interactive effect among the three factors. (DOCX) [file pone.0095196.s006.docx]

**Table S5** Summary of the partial General Linear Models (partial GLM) for the effects of PGF, climate and soil nutrient on leaf stoichiometric traits. *a, b,* and *c* denote the independent effect of plant growth form (PGF), climate and soil, respectively; *ab, ac,* and *bc* are respectively the interactive effect between PGF and climate, PGF and soil, climate and soil; *abc* denotes the interactive effect among the three factors

|  | Total effects (*R^2^*, %) | | | | Independent and interactive effects (*R^2^*, %) | | | | | | | unexplained (*R^2^*,%) |
| --- | --- | --- | --- | --- | --- | --- | --- | --- | --- | --- | --- | --- |
|  | Full | PGF (*a*) | Climate (*b*) | Soil (*c*) | *a* | *b* | *c* | *ab* | *ac* | *bc* | *abc* |  |
| C | 49.3 | 25.7 | 16.0 | 21.7 | 27.6 | 1.2 | 4.3 | -1.2 | 1.5 | 18.1 | -2.2 | 50.7 |
| N | 17.6 | 5.0 | 7.2 | 14.0 | 3.4 | 0.3 | 3.3 | 0.0 | 3.7 | 9.0 | -2.1 | 82.4 |
| P | 44.4 | 20.4 | 18.0 | 16.9 | 25.6 | 4.6 | 0.2 | -2.6 | 0.7 | 19.3 | -3.2 | 55.6 |
| C:N | 27.6 | 11.0 | 9.7 | 18.4 | 9.0 | 0.8 | 4.1 | -0.6 | 4.8 | 11.8 | -2.3 | 72.4 |
| C:P | 52.1 | 25.2 | 21.1 | 19.8 | 30.7 | 4.4 | 0.1 | -2.8 | 0.2 | 22.2 | -2.8 | 47.9 |
| N:P | 37.3 | 11.8 | 8.5 | 17.4 | 19.7 | 0.0 | 13.5 | 0.2 | -4.3 | 12.0 | -3.8 | 62.7 |
