# Supplementary material for: The Altitudinal Patterns of Leaf C∶N∶P Stoichiometry Are Regulated by Plant Growth Form, Climate and Soil on Changbai Mountain, China
Source: PLoS One. 2014 Apr 17;9(4):e95196. doi: 10.1371/journal.pone.0095196 (PMC3990608; doi:10.1371/journal.pone.0095196)
Supplement: Table S6 — Nutrition limitations of different vegetation types on Changbai Mountain. Nutrient limitations is diagnosed following criteria proposed by Koerselman (1996): If N∶P ratio >16, plant growth is limited by P availability. If N∶P ratio <14, plant growth is limited by N availability. If N∶P ratio between 14 and 16, plant growth is co-limited by N and P together. “N” indicates N limitation; “N and P” indicates N and P co-limitation. (DOCX) [file pone.0095196.s007.docx]

**Table S6** Nutrition limitations of different vegetation types on Changbai Mountain. Nutrient limitations is diagnosed following criteria proposed by Koerselman (1996): If N:P ratio >16, plant growth is limited by P availability. If N:P ratio <14, plant growth is limited by N availability. If N:P ratio between 14 and 16, plant growth is co-limited by N and P together. “N” indicates N limitation; “N and P” indicates N and P co-limitation

| Vegetation types |  | N:P ratio | | Limited element |
| --- | --- | --- | --- | --- |
|  | n | Mean | CV |  |
| Broad-leaved forest | 72 | 10.85 | 0.32 | N |
| Mixed coniferous broad- leaved forest | 91 | 11.50 | 0.23 | N |
| Dark-coniferous spruce-fir forest | 36 | 9.17 | 0.29 | N |
| Ermans birch forest | 38 | 12.60 | 0.21 | N |
| Alpine tundra | 22 | 14.55 | 0.24 | N and P |
| Alpine tundra | 20 | 13.80 | 0.18 | N |
